# Heritable gene editing using *FT* mobile guide RNAs and DNA viruses

**DOI:** 10.1186/s13007-021-00719-4

**Published:** 2021-02-17

**Authors:** Jianfeng Lei, Peihong Dai, Yue Li, Wanqi Zhang, Guantong Zhou, Chao Liu, Xiaodong Liu

**Affiliations:** College of Agriculture, Xinjiang Agricultural University, Engineering Research Centre of Cotton, Ministry of Education, 311 Nongda East Road, Urumqi, 830052 P.R. China

**Keywords:** CLCrV, CRISPR/Cas9, *FT*-sgRNA, VIGE

## Abstract

**Background:**

The virus-induced genome editing (VIGE) system can be used to quickly identify gene functions and generate knock-out libraries as an alternative to the virus-induced gene silencing (VIGS). Although plant virus-mediated VIGE has been shown to have great application prospects, edited genes cannot be transferred to the next generations using this system, as viruses cannot enter into shoot apical meristem (SAM) in plants.

**Results:**

We developed a novel cotton leaf crumple virus (CLCrV)-mediated VIGE system designed to target *BRI1*, *GL2*, *PDS* genes, and *GUS* transgene in *A. thaliana* by transforming Cas9 overexpression (Cas9-OE) *A. thaliana*. Given the deficiency of the VIGE system, *ProYao*::*Cas9* and *Pro35S*::*Cas9 A. thaliana* were transformed by fusing 102 bp *FT* mRNAs with sgRNAs so as to explore the function of Flowering Locus T (*FT*) gene in delivering sgRNAs into SAM, thus avoiding tissue culture and stably acquiring heritable mutant offspring. Our results showed that sgRNAs fused with *FT* mRNA at the 5′ end (*FT* strategy) effectively enabled gene editing in infected plants and allowed the acquisition of mutations heritable by the next generation, with an efficiency of 4.35–8.79%. In addition, gene-edited offspring by *FT*-sgRNAs did not contain any components of the CLCrV genome.

**Conclusions:**

*FT* strategy can be used to acquire heritable mutant offspring avoiding tissue culture and stable transformation based on the CLCrV-mediated VIGE system in *A. thaliana*.

## Background

The acquisition of heritable mutations is essential for identifying gene functions and breeding new crop varieties. Genome editing is a powerful tool that induces DNA double-strand breaks (DSBs) at specific genomic loci, leading to targeted mutations by triggering DNA damage repair mechanisms [1]. Unlike first-generation genome editing tools, Type II CRISPR/Cas9 genome editing, which has been widely applied for crop improvement, involves simple designing and cloning methods [2–6]. During this process, single guide RNAs (sgRNAs) is used to guide the endonuclease Cas9 to cut a double-stranded DNA (dsDNA) at precise target loci. This process leads to DSBs repair through nonhomologous end-joining (NHEJ) or homologous recombination (HR). In the absence of homologous DNA templates, the repair of DSBs through NHEJ may cause deletions, insertions, or substitutions of bases at target loci, resulting in heritable variations at specific loci [7]. However, the application of CRISPR/Cas9 in plants may be challenging because the acquisition of heritable mutations via CRISPR/Cas9 usually requires a pathway of stable transformation, which is both time-consuming and laborious.

Recent studies have found that sgRNAs, a key component in the CRISPR system, are inefficient or invalid at many genomic loci. In addition, their efficiency directly impacts the application of CRISPR/Cas9 in plants, and their activity may not be predicted by bioinformatics methods [8, 9]. Therefore, before acquiring mutations using the CRISPR system, it is necessary to minimize the risk of using invalid sgRNAs, which requires prompt screening of efficient sgRNAs. Protoplast transformation with intact gene editing vectors [10] and *Agrobacterium*-mediated transient transformation of plant leaves with intact gene editing vectors [11] are the two commonly used methods to identify the activity of sgRNAs. Nevertheless, these methods have a high failure frequency.

Recent studies have shown that sgRNAs can be delivered in plants by using plant viruses, also known as the virus-induced genome editing (VIGE) [12, 13]. This method offers a few advantages compared to other conventional ways of transforming intact gene editing vectors. Firstly, sgRNAs can be produced, reproduced, spread, and accumulated along with virus in the whole plant, thus inducing target gene editing mutations in more cells and significantly improving the detection rate of gene editing. Secondly, VIGE can assemble multiple sgRNAs on one viral genome to enable multiplexed gene editing. Finally, VIGE can identify gene functions without stable transformation. Some RNA viruses, including tobacco rattle virus (TRV) [14], pea early-browning virus (PEBV) [15], tobacco mosaic virus (TMV) [16], and beet necrotic yellow vein virus (BNYVV) [17], have been used for targeted editing in model plants, such as *A. thaliana* and *N. benthamiana*. Also, the barley stripe mosaic virus (BSMV)-mediated VIGE system was established in hexaploid wheat [12]. Plant DNA virus-mediated VIGE was also developed and used afterward [13]. Although plant virus-mediated VIGE has shown great application prospects, a CLCrV-mediated VIGE system has not yet been reported.

Viruses can efficiently deliver sgRNAs to enable the editing of target genes. Nevertheless, such editing events can only occur in the somatic cells of infected plants. Edited genes cannot be transferred to the next generations since a special mechanism makes it difficult for viruses to enter into their SAM, necessary to edit germ cells in plants [13, 18, 19]. Gene editing in SAM is required for the acquisition of heritable mutant offspring in the VIGE system. A previous study has demonstrated that Flowering Locus T (*FT*) mRNA enabled the entry of heterologous RNA fused with it into SAM [20]. Therefore, in this study, we proposed a new *FT* strategy that enables the fusion of sgRNAs with *FT* mRNA, allowing the sgRNAs to enter SAM. This process eventually breaks the deficiency of the stable transformation pathway limit and acquire gene-edited offspring.

Cotton leaf crumple virus (CLCrV) is a two-component DNA virus composed of the CLCrV-A genome and CLCrV-B genome. Existing studies have shown that CLCrV as a virus-induced gene silencing (VIGS) vector, can be used to silence the expression of endogenous genes in plants [21]. To this end, we developed a CLCrV-mediated VIGE system and successfully acquired gene-edited offspring in *A. thaliana* using the *FT* strategy.

## Methods

### Plant materials and growth conditions

*A. thaliana* Col-0 ecotype plants were transformed by the floral dipping method as previously described [22]. For the selection of transformants, seeds were sterilized with 5% NaClO for 5 min and plated on 1/2 MS medium [23] with 30 μg/mL kanamycin. After 2 weeks, resistant seedlings were transplanted to the soil under long-day conditions (16 h light/8 h dark) at 22 °C. Homozygous transgenic seedlings grown for about 15 days were used for inoculation transformation experiments.

### Vectors construction

To construct the 35S::*Cas9*-Ter-P2301 vector, the 35S::*Cas9*-Ter fragments (5476 bp) were recovered from 35S::*Cas9*-Ter-SK vector [24] digested with *Hind*III and *Xba*I, and recombined into pCAMBIA2301 (with a *GUS* gene in the vector) to obtain 35S::*Cas9*-Ter-P2301 vector. Then, 10 μL 35S::*Cas9*-Ter-P2301 plasmid was introduced into *A. tumefaciens* strain GV3101. Transformed *Agrobacterium* cultures were grown at 28 °C for 2 days, and positive clones were selected and cultured in LB medium (containing 50 μg/mL kanamycin and 50 μg/mL rifampicin) to the logarithmic growth stage for *A. thaliana* genetic transformation.

To construct the sgRNA vector, a truncated AtU6-26 (330 bp) promoter [24] was used to drive sgRNA expression. The 20 bp guide RNA (Additional file 1) was inserted into AtU6-26::sgRNA vector digested with *Bbs*I. Sequencing was used to verify whether the sgRNA vector containing the target sequence was correct. The CLCrV-A coat protein gene was replaced by a multi-cloning site to facilitate the assembly of sgRNA. Different sgRNA vectors were digested with *Spe*I and *Pac*I, and ligated into CLCrV-A vector. The recombinant plasmids were verified by the restriction enzyme digestion method.

To construct the *FT*-sgRNA vector, *A. thaliana* Col-0 ecotype cDNA and PCR primers (Additional file 1) were used to clone *FT* (528 bp) gene. Then, 102 bp *FT* was fused to the 5′ end of *AtBRI1*-sgRNA by transfer PCR [25]. Sequencing was used to verify whether the *FT*- *AtBRI1*-sgRNA vector was correct.

### RT-PCR analysis of *Cas9* expression

Total RNA was isolated from Cas9-OE *A. thaliana* and reversely transcribed into cDNA. *A. thaliana Atactin2* gene was used as the reference gene. RT-PCR was performed at 94 °C for 3 min followed by 27 cycles of amplification (94 °C for 30 s, 60 °C for 30 s and 72 °C for 30 s) and a final extension of 10 min at 72 °C. According to the template amount of the reference gene, the target *Cas9* fragment was amplified. Primers used for PCR amplification are listed in Additional file 1.

### Transient transformation in Cas9-OE *A. thaliana* with CLCrV-sgRNA

For transient expression and virus inoculation, all CLCrV-AtU6-26::sgRNA vectors and CLCrV-B were introduced into *A. tumefaciens* strain GV3101. The *Agrobacterium* cultures were inoculated in a 5 mL LB medium (containing 50 μg/mL kanamycin and 50 μg/mL rifampicin) and grown overnight in a 28 °C shaker. *Agrobacterium* cultures were harvested and resuspended in infiltration buffer (10 mM MgCl_2_, 10 mM MES and 200 μM acetosyringone), adjusted to an optical density at 600 nm of 1.0, and incubated at room temperature for 3 to 4 h. For virus inoculation, *Agrobacterium* cultures containing CLCrV-B and CLCrV-A or their derivatives were mixed at a 1:1 ratio. The mixed *Agrobacterium* solutions were infiltrated into *A. thaliana* leaves with a 1 ml syringe.

### GUS staining and quantitative analysis

The upper systemic leaves without injection were GUS stained after infiltration according to a previously described protocol [26]. Briefly, to analyze *GUS* gene expression, RNA was isolated from the upper systemic leaves of plant inoculated CLCrV-A and CLCrV-B empty vector, *GUS*-sgRNA and Cas9-OE *A. thaliana*, and reversely transcribed into cDNA. qRT-PCR was performed amplifying the 168 bp *GUS* gene. After the reaction was completed, relative gene expression levels were calculated using the 2^−∆∆Ct^ method [27] with *A. thaliana Actin2* gene as the reference gene. Primers used for PCR amplification are listed in Additional file 1.

### Mutation detection

For target gene editing detection, genomic DNA was isolated from *A. thaliana* leaves inoculated with CLCrV-B and CLCrV-A derivatives. PCR/RE assay was performed according to previously described methods [11]. Briefly, PCR amplified a genomic fragment containing the target site, and appropriate restriction enzymes were used to digest PCR products in order to confirm mutations at the target site. The undigested PCR amplicons were cloned into a Blunt Zero cloning vector and sequenced. All primers used for PCR amplification are listed in Additional file 1.

### Statistical analyses

Statistical analysis of the numerical data was performed using Excel 2013 and SPSS 18.0. For multiple pairwise comparisons, the data were compared using a one-way ANOVA test. * *p* < 0.05 was considered statistically significant, and ** *p* < 0.01 was considered extremely significant.

## Results

### Design of CLCrV-mediated sgRNA delivery system

**Fig. 1:**
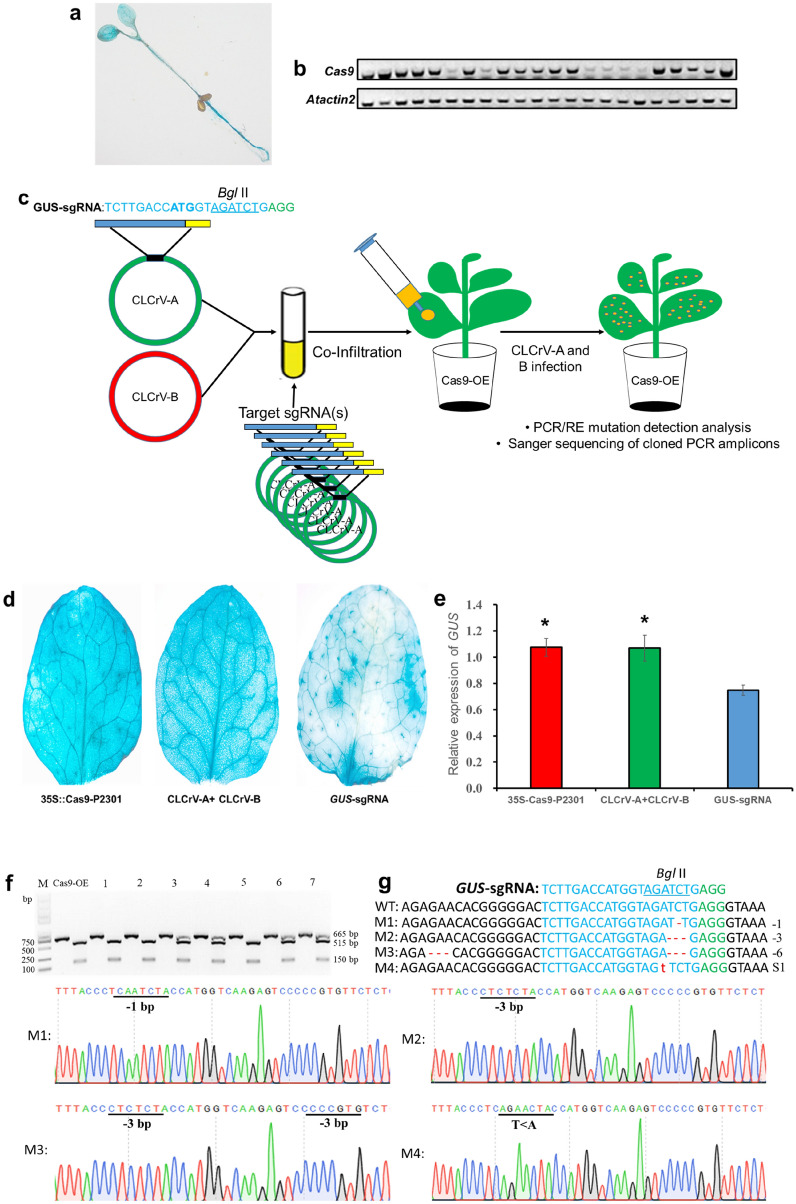
*GUS* reporter gene system targeted by CLCrV-mediated VIGE. **a** GUS staining of Cas9-OE *A. thaliana* seedling. **b** *Cas9* expression levels in transgenic plants determined by RT-PCR. **c** Experimental scheme of the CLCrV-mediated genome editing. **d** GUS staining in Cas9-OE *A. thaliana* leaves infiltrated with *Agrobacterium* carrying different constructs. GUS staining of leaves inoculated with CLCrV-A and CLCrV-B empty vectors that served as a control, the blue area of leaves inoculated with *GUS*-sgRNA decreased. **e** *GUS* expression levels determined by qRT-PCR. Asterisks indicate significant differences (* *p* < 0.05). **f** and **g** Detection of *GUS*-sgRNA targeted mutations. **f** Cas9-OE plant served as a control, 1–7 were plant numbers. The gel image shows PCR products of the *GUS* gene, and digested PCR products with *Bgl*II. **g** The undigested PCR products lacking the *Bgl*II site (due to the presence of a mutation) that were subsequently purified, cloned, and analyzed by sequencing. The green color indicated the PAM sequence. The *Bgl*II restriction site on the target sequence is underlined in blue. M indicates the mutation sequence. Deletions are shown in red dashes. Substitutions are denoted with red lowercase letters

The feasibility of CLCrV-mediated VIGE was analyzed in *A. thaliana* to determine whether the geminivirus CLCrV could be used to deliver sgRNAs for targeted genes in plants. The high expression of *Cas9* and sgRNAs is the key step in the acquisition of a high mutation rate [28, 29]. Considering the size of *Cas9*, transforming cells with an intact CRISPR genome editing vector may be challenging and usually not enough to enable valid targeted editing. Thus, in this study, we established a transgenic Cas9-OE *A. thaliana* in which *GUS* is overexpressed before delivering sgRNAs using CLCrV. Transgene-positive plants were blue when they were GUS stained (Fig. 1a). RT-PCR was further used to verify the valid expression of *Cas9* in transgenic plants (Fig. 1b). Transgenic plants with higher *Cas9* expressions were used in later studies.

The GUS reporter system was used to detect the efficiency of the CLCrV-mediated VIGE system. A guide RNA was designed to target *Bgl*II site downstream start codon on the *GUS* gene. The CLCrV-AtU6-26::*GUS*-sgRNA was co-transformed into the leaves of Cas9-OE *A. thaliana* with CLCrV-B (Fig. 1c). The blue zones in leaves were significantly narrowed, while the corresponding leaves were infiltrated with the CLCrV vector without sgRNA remained blue, which was similar to that observed in transgenic GUS plants (Fig. 1d). Furthermore, the qRT-PCR proved the decrease of *GUS* gene expression in plants infiltrated with *GUS*-sgRNA (Fig. 1e).

The PCR/RE analysis confirmed the mutations at *Bgl*II site in the *GUS* gene in *A. thaliana* infiltrated with *GUS*-sgRNA, which was not observed in the control group (Fig. 1f). Sequencing results showed mutations, including − 1 bp, − 3 bp, and − 6 bp deletions and base substitutions in the target sequence region (Fig. 1g). Among them, − 1 bp deletion caused the region's codon to mutate to the stop codon TGA, resulting in early *GUS* gene translation termination. The above results demonstrated that delivery of CLCrV-mediated sgRNA into the Cas9-OE plants could effectively induce targeted gene editing in plants.

### CLCrV-mediated targeted mutagenesis in endogenous genes in *A. thaliana*

**Fig. 2:**
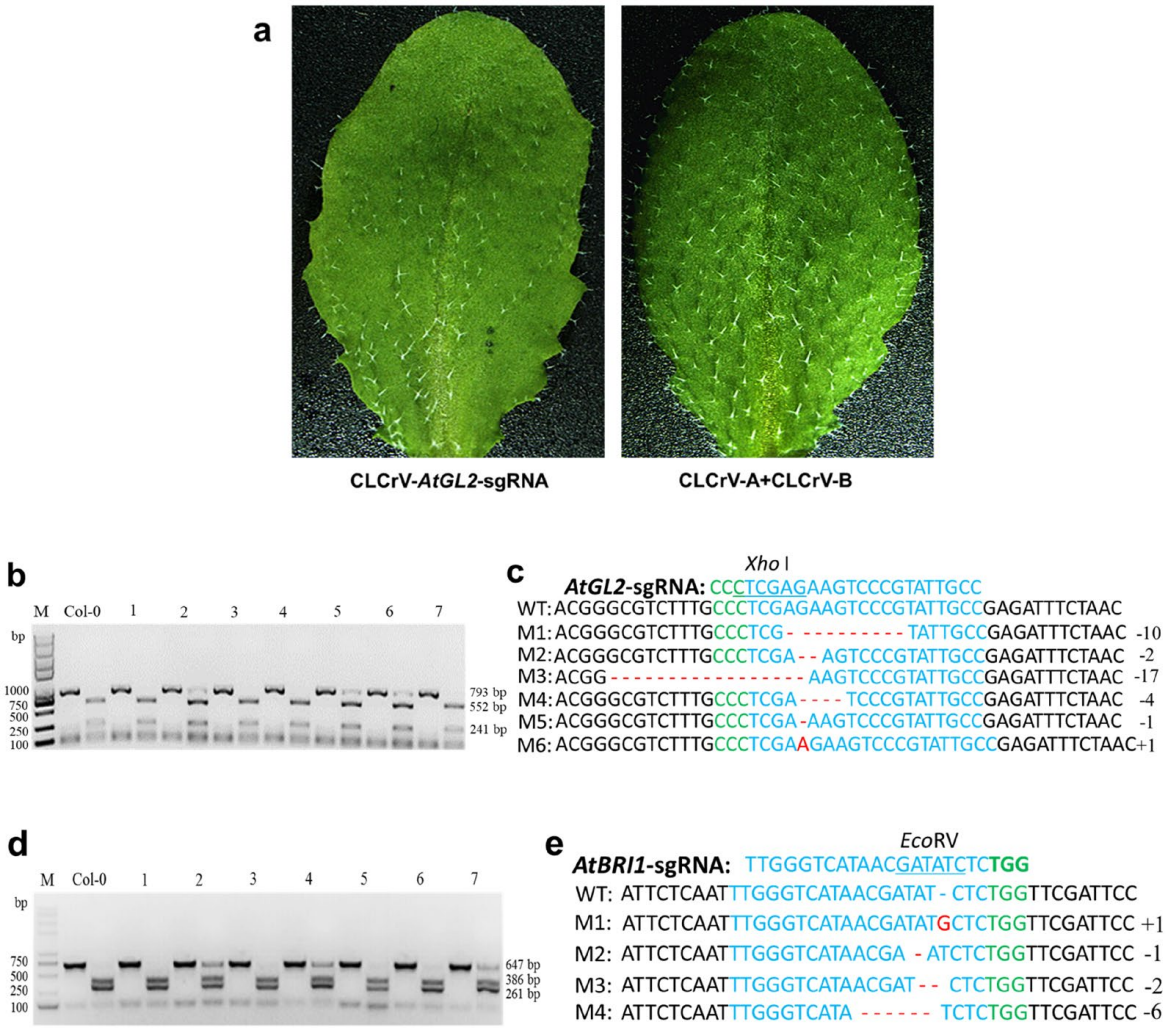
CLCrV-mediated targeted mutagenesis of *AtGL2* and *AtBRI1* in *A. thaliana*. **a** Mutant phenotype of systemically infected Cas9-OE *A. thaliana* leaves at 15–25 days post-infiltration with CLCrV-*AtGL2*-sgRNA. *A. thaliana* plants inoculated with CLCrV-A and CLCrV-B empty vectors served as a control. **b** and **c** Detection of *AtGL2*-sgRNA targeted mutations. **b** Col-0 served as a control, 1–7 were plant numbers. The gel image shows PCR products of the *AtGL2* gene and digested PCR products with *Xho*I. **c** The undigested PCR products lacking the *Xho*I site (due to the presence of a mutation) that were subsequently purified, cloned, and analyzed by sequencing. **d** and **e** Detection of *AtBRI1*-sgRNA targeted mutations. **d** Col-0 served as a control, 1–7 were plant numbers. The gel image shows PCR products of the *AtBRI1* gene and digested PCR products with *Eco*RV. **e** The undigested PCR products lacking the *Eco*RV site (due to the presence of a mutation) that were subsequently purified, cloned, and analyzed by sequencing. The green color indicated the PAM sequence. The restriction site on the target sequence is underlined in blue. M indicates the mutation sequence. Deletions are shown in red dashes. Insertions are denoted with red capital letters

In order to determine whether CLCrV-mediated VIGE was capable of targeted editing of endogenous genes in *A. thaliana* and causing mutation phenotypes, *BRI1-*gene modulating development [30] and *GL2* gene-regulating trichome development in *A. thaliana* [31] were used as targets. CLCrV-AtU6-26::*AtBRI1*-sgRNA and CLCrV-AtU6-26::*AtGL2*-sgRNA were injected into the leaves of Cas9-OE *A. thaliana*, respectively. The leaves transformed with CLCrV-AtU6-26::*AtGL2*-sgRNA exhibited fewer trichomes compared with empty CLCrV vectors (Fig. 2a), while the leaves transformed with CLCrV-AtU6-26::*AtBRI1*-sgRNA showed no mutation phenotypes. Mutations in *AtGL2* and *AtBRI1* genes were further detected by PCR/RE and sequencing. Results showed that the gene mutations were base deletions or insertions in the target sequence region (Fig. 2b-e and Additional files 2, 3). We observed no *BRI1* mutation phenotype, probably because the edited cell's phenotype was covered by the normal cell. The system also enabled targeted editing of the *PDS* gene in *A. thaliana* (Additional file 4). As indicated by our experimental results, CLCrV-mediated VIGE could be used to enable targeted editing of endogenous genes in *A. thaliana*, which was useful for studying gene functions.

### Tissue-specific Cas9 system improving gene editing efficiency

*Cas9* gene driven by tissue-specific promoters could effectively improve editing efficiency [32]. To improve the mutation efficiency of CLCrV-mediated VIGE, we used *ProYao*::*Cas9* transgenic *A. thaliana* in which *Cas9* is preferentially expressed in meristem as the transformation receptor [32]. *AtBRI1* and *AtGL2* genes were used as targets, PCR/RE and sequencing results showed that the editing efficiency of *ProYao*::*Cas9* in *AtBRI1* and *AtGL2* genes were significantly higher (50.00% and 62.50%, respectively) than that of *Pro35S*::*Cas9* (18.75% and 18.75%, respectively) (Table 1 and Additional file 5). Results indicated that *ProYao*::*Cas9* could be used as an ideal receptor for CLCrV-mediated VIGE.

**Table 1:** Comparison of mutation frequency between *Pro35S*::*Cas9* and *ProYao*::*Cas9* in CLCrV-mediated VIGE system in *A. thaliana*

Transgenic receptor	Target	Mutation frequency (%)
*ProYao*::*Cas9*	*AtBRI1*-sgRNA	50.0% (8/16)
	*AtGL2*-sgRNA	62.5% (10/16)
*Pro35S*::*Cas9*	*AtBRI1*-sgRNA	18.75% (3/16)
	*AtGL2*-sgRNA	18.75% (3/16)

### Heritable gene editing using *FT* mobile guide RNAs

**Fig. 3:**
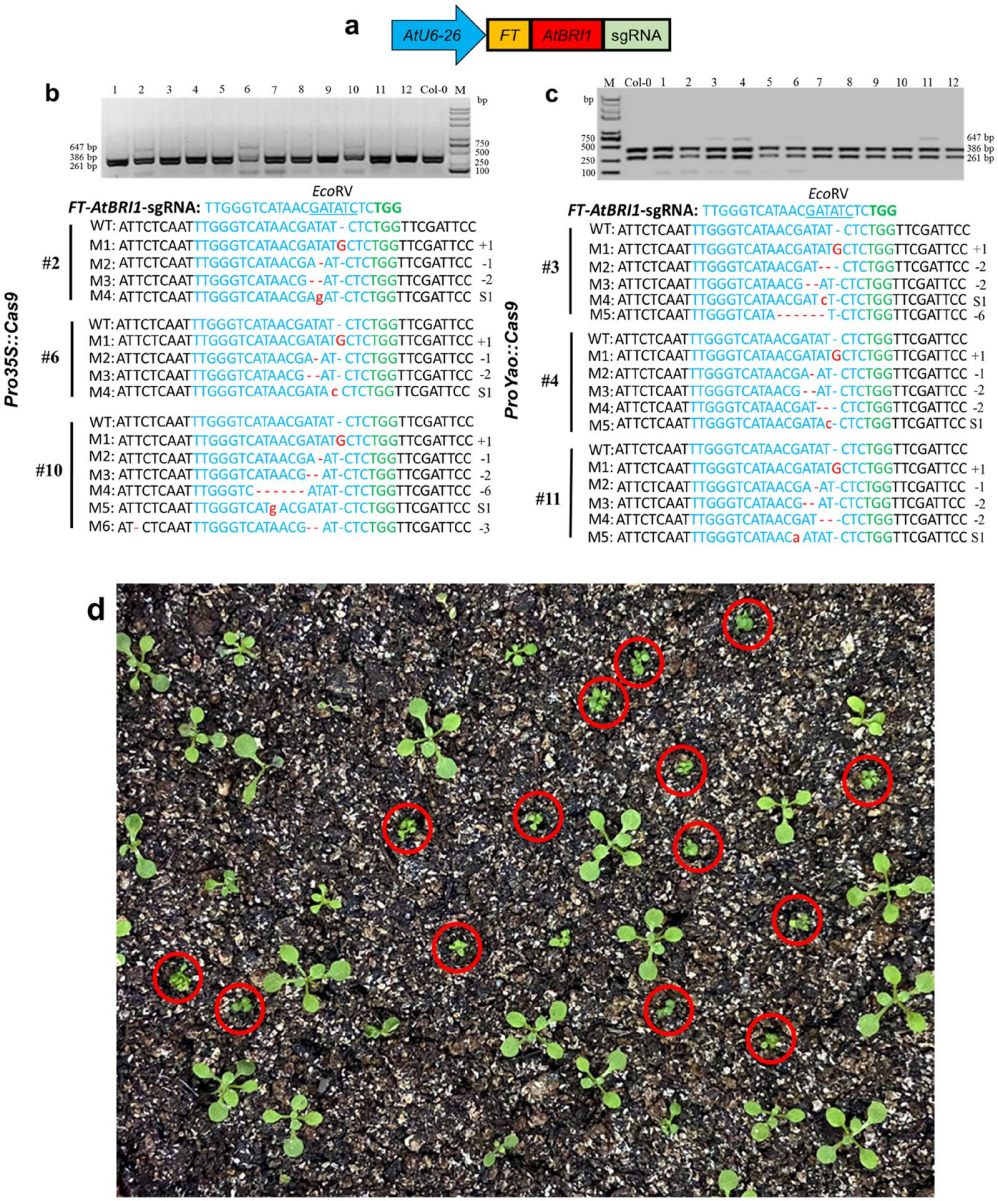
*FT*-sgRNA targeted mutagenesis of *AtBRI1* in *A. thaliana*. **a** Truncated *FT* RNA was fused to the 5′ end of *AtBRI1*-sgRNA. **b** and **c** Detection of *FT-AtBRI1-*sgRNA targeted mutations in *Pro35S*::*Cas9* and *ProYao*::*Cas9 A. thaliana*. Col-0 served as a control, 1–12 were plant numbers. The gel image shows digested PCR products of the *AtBRI1* gene with *Eco*RV, the undigested PCR products lacking the *Eco*RV site (due to the presence of a mutation) that were subsequently purified, cloned, and analyzed by sequencing. The green color indicated the PAM sequence. The restriction site on the target sequence is underlined in blue. M indicates the mutation sequence. Deletions are shown with red dashes. Insertions are denoted with red capital letters. Substitutions are denoted with red lowercase letters. **d** Phenotype of *bri1* mutations in M2 generation. The red circle shows the *bri1* mutant

Flowering Locus T (*FT*) gene has an important role in inducing flowering in *A. thaliana* [33–36]. *FT* mRNA could systematically diffuse in plants and migrate to the SAM of plants independent of *FT* proteins. *FT* encoding sequences from the initiation codon to the 102^nd^ nucleotides were responsible for *FT* mobility [37, 38], which can take the heterologous RNA fused with it into SAM [20]. Therefore, we investigated whether *FT* mRNA could deliver sgRNAs to SAM, causing editing events in meristem and finally acquiring stably heritable mutations without stable transformation. The feasibility of this approach was verified in *A. thaliana*. *AtBRI1* gene of *A. thaliana* was used as the target. *FT* (102 bp) gene was fused to the 5′ end of *AtBRI1* sgRNA since the first 102 bp *FT* segments were responsible for *FT* mRNA mobility (Fig. 3a). The leaves of *Pro35S*::*Cas9* and *ProYao*::*Cas9 A. thaliana* were transformed with *FT*-sgRNA expression vector-mediated with *A. tumefaciens*. Results showed that AtU6-26::*FT*-*AtBRI1*-sgRNA caused editing event in infiltrated leaves of *Pro35S*::*Cas9* plants (#2, #6 and #10) and *ProYao*::*Cas9* (#3, #4 and #11) (Fig. 3b, c and Additional file 6), which in turn indicated that the fusion of 102 bp *FT* mRNA to the 5′ end of sgRNA could also effectively enable gene editing.

To test whether *FT* mRNA takes sgRNA into SAM and leads to the mutation in a germ cell, seeds of the progenies of Cas9-OE plants with mutation made by *FT*-sgRNA and unmodified sgRNA were sown. No phenotype was observed in the offspring (M1) of transformed *FT*-*AtBRI1*-sgRNA and unmodified sgRNA plants. However, PCR/RE and sequencing analysis was performed on M1 plants, revealing *AtBRI1* gene mutations in some offspring of transformed *FT*-*AtBRI1*-sgRNA. The mutation efficiency of *AtBRI1* in the M1 generation ranged from 4.35 to 8.79%, no significant difference was observed between *Pro35S*::*Cas9* plants and *ProYao*::*Cas9* plants. Sequencing results showed that most of the mutant plants contained only two genotypes (deletions of -AT and WT, and insertions of + G and WT) and were presumed to be heterozygotes. In comparison, only a few of them contained two or more genotypes and were presumed to be chimeras. No *AtBRI1* mutations were detected in the offspring of transformed unmodified sgRNA plants (Table 2). The seeds (M2 generation) from the mutant plants (M1) were sown and transplanted into the soil, and the *BRI1* mutation phenotype was obviously present in some plants within two weeks (Fig. 3d). It was inferred that mutations were inherited in the form of heterozygotes according to statistical results of the phenotypic segregation ratio of the M2 generation of *BRI1* (Table 3) and the Chi-squared Test result of 3:1. The results showed that the *FT*-sgRNA strategy in *A. thaliana* could cause heritable mutations in the form of heterozygotes, while homozygotes could only be acquired in the M2 generation.

**Table 2:** Mutation efficiency of *AtBRI1* gene in system leaf and transgenic M1 generation

Transgenic receptor	Modification	Target	System leaf mutation frequency of M0	Plant number	No. of M1 edited plants showing excepted phenotype	No. of M1 edited plants	Mutation frequency of M1
*Pro35S*:*:Cas9*	Unmodified sgRNA	*BRI1*-sgRNA	(3/12) 25%	#2	N/A	0	0.00%(0/68)
				#4	N/A	0	0.00%(0/48)
				#7	N/A	0	0.00%(0/72)
	*FT*-sgRNA	*FT*-*BRI1*-sgRNA	(3/12) 25%	#2	N/A	3	4.35%(3/69)
				#6	N/A	4	8.33%(4/48)
				#10	N/A	8	8.79%(8/91)
*ProYao*:*:Cas9*	Unmodified sgRNA	*BRI1*-sgRNA	(8/16)50%	#12	N/A	0	0.00%(0/44)
				#13	N/A	0	0.00%(0/60)
				#14	N/A	0	0.00%(0/64)
	*FT*-sgRNA	*FT*-*BRI1*-sgRNA	(3/12) 25%	#3	N/A	3	6.52%(3/46)
				#4	N/A	5	8.06%(5/62)
				#11	N/A	3	5.56%(3/54)

**Table 3:** Genetic analysis of *BRI1* mutations in M2 generation

Transgenic receptor	Plant number	Total number of plants	No. of M2 edited plants	No. of M2 no edited plants	χ^2^
*Pro35S*:*:Cas9*	#2–6	52	12	40	0.0256
	#2–10	68	14	54	0.4902
	#2–12	49	11	38	0.0612
*ProYao*:*:Cas9*	#4–5	79	18	61	0.1055
	#4–10	71	14	57	0.7934
	#4–36	47	10	37	0.1773

The table above shows the statistical analysis results of effects of unmodified sgRNAs and *FT*-sgRNA vectors on mutation efficiency of system leaf and M1 generation at *AtBRI1* target loci. N/A indicates the absence of an obvious phenotype.

### Virus detection in mutant offspring (M1)

**Fig. 4:**
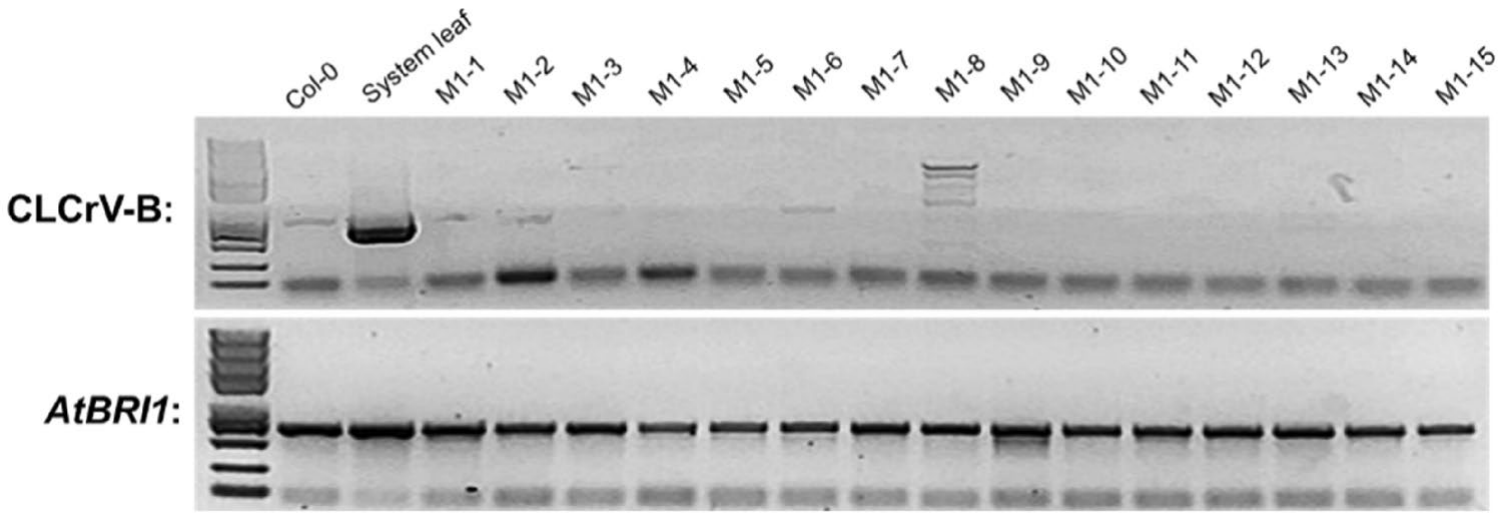
Detection of virus accumulation in M1 generation

In order to detect whether CLCrV is transmitted to the next generation, PCR amplification of CLCrV-B genome and *AtBRI1* gene were performed using DNA samples isolated from *FT*-*AtBRI1*-sgRNA (#2) system leaf and part of its M1 generation. Results showed that except for the detection of virus accumulation in system leaves, no virus accumulation was detected in the M1 generation (Fig. 4).

## Discussion

The broad application prospects of gene editing systems, such as identifying gene functions and molecular design breeding, have been extensively applied in plants. Yet, this approach requires a complete process of stable transformation, i.e., tissue culture in most plants, which is time-consuming, thus making it impossible to identify gene functions on a large scale. A recently developed virus-mediated gene editing system (VIGE) [12, 13] does not require tissue culture because sgRNA expression is accompanied by the replication, accumulation, and spread of viruses. This new method can induce editing events in almost the whole plant, causing an obvious phenotype due to defective genes.

Cotton leaf crumple virus (CLCrV) is a DNA virus whose infection is not affected by coat protein deficiency [39]. The virus can carry 800 bp of exogenous DNA segments for gene silencing [21]. SgRNA and Cas9 are two essential components of the CRISPR/Cas9 system, Cas9 does not change regardless of which locus of a genome is edited. The carrying capacity of CLCrV is enough for sgRNA expression, although not suitable for Cas9 protein expression. Therefore, in this study, we designed a new system in which the Cas9 overexpression line was used as a transformation receptor, coat protein genes of CLCrV were replaced with sgRNAs, and transcription of sgRNAs was driven by truncated AtU6-26 (330 bp) promoters [24]. This approach only required CLCrV to deliver sgRNAs, thus avoiding transforming intact editing vectors. By using the CLCrV-mediated delivery system, the *GUS* gene in Cas9 transgenic *A. thaliana* was successfully targeted, which proves that CLCrV could be useful in delivering sgRNAs (Fig. 1) and enabling targeted editing of endogenous genes in *A. thaliana* (Fig. 2).

Previous studies have reported that heritable editing events are recovered in the TRV-mediated VIGE system with a very low genome-editing efficiency [14]. Still, the CLCrV-mediated sgRNA delivery system fails to cause heritable mutations in *A. thaliana* compared with the TRV-mediated VIGE system in *N. benthamiana*. CLCrV-mediated VIGE cannot occur in SAM and can only exist in somatic cells due to the virus's limited replication [40]. Therefore, we further explored the function of the Flowering Locus T (*FT*) gene in *A. thaliana* to deliver sgRNAs to the SAM of plants. We fused *FT* mRNA to the 5′ end of sgRNA and transiently transforming *Pro35S*::*Cas9* and *ProYao*::*Cas9* transgenic *A. thaliana*. Our data showed that *FT*-sgRNA has the same editing efficiency as unmodified sgRNA in the infected plant and can pass on the mutation to offspring. In addition, gene-edited offspring by *FT*-sgRNAs contains no components of the CLCrV genome (Fig. 4). Compared to *Pro35S*::*Cas9*, the CLCrV-mediated VIGE system had no apparent advantage in heritable gene editing frequencies in *ProYao*::*Cas9* transgenic *A. thaliana* (Table 2), although it showed higher editing efficiency in the infected plant (Table 1). It was recently reported that *PDS* mutations were acquired in the offspring of *N. benthamiana* without tissue culture by fusing *FT* mRNAs to the 3′ end of *PDS* sgRNAs and assembling the construction in RNA virus TRV vectors, which were transformed in Cas9-OE *N. benthamiana*. The highly-efficient mutant acquisition proved that *FT* mRNAs could deliver the RNAs fused with it to the SAM of plants over long distances, thus significantly improving the application efficiency of gene-editing technology in the plant [41]. Our results also showed that *FT* mRNA was capable of delivering sgRNAs to the SAM of plants, which could acquire heritable mutant offspring when fused *FT* mRNAs to the 5′ end of sgRNAs. Our results also proved that the *FT*-sgRNA strategy could work, although the efficiency of *FT-*sgRNA gene editing mediated by CLCrV (4.35% to 8.79%) was much lower than that mediated by TRV (65% to 100%). Based on the fact that the movement efficiency of *FT*-RNAs to SAM did significantly differ between different plant species [42, 43], we assumed that the mutant progeny is lower, possibly because of the inefficient mobility of *FT* mRNAs to SAM in *A. thaliana* [42].

## Conclusions

CLCrV-mediated VIGE enables efficient gene editing in *A. thaliana*. The *FT*-sgRNA strategy was successfully applied in *A. thaliana*, thus suggesting its broad application prospects in functional genomics and molecular design breeding in crops.

## Supplementary Information

**Additional file 1.** Primer sequences involved in this study.

**Additional file 2. **The DNA sequence of targeted editing of *AtGL2* by *AtGL2*-sgRNA.

**Additional file 3. **The DNA sequence of targeted editing of *AtBRI1 *by *AtBRI1*-sgRNA.

**Additional file 4. **CLCrV-mediated targeted knockout of *AtPDS*.

**Additional file 5. **Tissue-specific Cas9 system for mutation detection of *AtBRI1* and *AtGL2*.

**Additional file 6. **The DNA sequence of targeted editing of *AtBRI1* by *FT*-*AtBRI1*-sgRNA.

## Data Availability

The authors are pleased to share analyzed/raw data and plant materials upon reasonable request.
