# Supplementary material for: Heritable gene editing using FT mobile guide RNAs and DNA viruses
Source: Plant Methods. 2021 Feb 17;17:20. doi: 10.1186/s13007-021-00719-4 (PMC7890912; doi:10.1186/s13007-021-00719-4)
Supplement: Supplementary file 1 — Additional file 1. Primer sequences involved in this study. [file 13007_2021_719_MOESM1_ESM.docx]

**Additional Files**

**Heritable gene editing using *FT* mobile guide RNAs and DNA viruses**

Jianfeng Lei, Peihong Dai, Yue Li, Wanqi Zhang, Guantong Zhou, Chao Liu and Xiaodong Liu^*^

College of Agriculture, Xinjiang Agricultural University, Engineering Research Centre of Cotton, Ministry of Education, 311 Nongda East Road, Urumqi 830052, P.R. China

^*^Correspondence: Xiaodong Liu (xiaodongliu75@aliyun.com)

**Additional file 1.** Primer sequences involved in this study.

| Primer | Sequence(5'-3') | Application |
| --- | --- | --- |
| GUS-sgRNAF: | GATTGCTTGACCATGGTAGATCTG | Construction of sgRNA (GUS):5'-GCTTGACCATGGTAGATCTG-3' |
| GUS-sgRNAR: | AAACCAGATCTACCATGGTCAAGC |  |
| AtBRI1-sgRNAF: | GATTGTGGGTCATAACGATATCTC | Construction of sgRNA (*AtBRI1*):5'-GTGGGTCATAACGATATCTC-3' |
| AtBRI1-sgRNAR: | AAACGAGATATCGTTATGACCCAC |  |
| AtPDS-sgRNA1F: | GATTGAAATTGAGCTCAATGACGA | Construction of sgRNA1 (*AtPDS*):5'-GAAATTGAGCTCAATGACGA-3' |
| AtPDS-sgRNA1R: | AAACTCGTCATTGAGCTCAATTTC |  |
| AtPDS-sgRNA2F: | GATTGAGCAGAGGAATGGATATCA | Construction of sgRNA2 (*AtPDS*):5'-GAGCAGAGGAATGGATATCA-3' |
| AtPDS-sgRNA2R: | AAACTGATATCCATTCCTCTGCTC |  |
| AtPDS-sgRNA3F: | GATTGAGTACTTAGCTTCCATGGA | Construction of sgRNA3 (*AtPDS*):5'-GAGTACTTAGCTTCCATGGA-3' |
| AtPDS-sgRNA3R: | AAACTCCATGGAAGCTAAGTACTC |  |
| AtPDS-sgRNA4F: | GATTGCTCCAGATAGCTGCATGGA | Construction of sgRNA4 (*AtPDS*):5'-GCTCCAGATAGCTGCATGGA-3' |
| AtPDS-sgRNA4R: | AAACTCCATGCAGCTATCTGGAGC |  |
| AtGL2-sgRNAF: | GATTGGCAATACGGGACTTCTCGA | Construction of sgRNA (*AtGL2*):5'-GGCAATACGGGACTTCTCGA-3' |
| AtGL2-sgRNAR: | AAACTCGAGAAGTCCCGTATTGCC |  |
| M-GUSF: | AACCACGTCTTCAAAGCAAG | Amplification of a 665 bp DNA fragment flanking *GUS* target |
| M-GUSR: | TCCTGATTATTGACCCACAC |  |
| M-AtBRI1:F | GATGGGATGAAGAAAGAGTG | Amplification of a 647 bp DNA fragment flanking *AtBRI1* target |
| M-AtBRI1:R | CTCATCTCTCTACCAACAAG |  |
| M-AtPDS-1F: | GGCGCTAAACTTTATAAACCC | Amplification of a 684 bp DNA fragment flanking *AtPDS* target |
| M-AtPDS-1R: | CTGGTAAAAGGAGCTTCAGG |  |
| M-AtPDS-2F: | CTTGTAAGGTAAGAACGCGG | Amplification of a 741 bp DNA fragment flanking *AtPDS* target |
| M-AtPDS-2R: | GCAATTTCTCTACATAACCGG |  |
| M-AtPDS-4F: | ACTGCAAAGTACCTGGCTGA | Amplification of a 761 bp DNA fragment flanking *AtPDS* target |
| M-AtPDS-4R: | GATAAGCACCGACTTCCAAC |  |
| M-AtGL2-F: | AGTTAGGGTTCAGTTGCATG | Amplification of a 793 bp DNA fragment flanking *AtGL2* target |
| M-AtGL2-R: | ACTCTGGGCAAGTTTATGTG |  |
| AtFT-CDSF: | ATGTCTATAAATATAAGAGACCCTC | PCR amplification of *AtFT* (528 bp) |
| AtFT-CDSR: | CTAAAGTCTTCTTCCTCCGC |  |
| FT-BRI1F: | CTGAGTTTATATACAGCTAGAGTCGAAGTAGTGATTGATGTCTATAAATATAAGAGACCCTC | Transfer PCR amplification of *FT*-*BRI1-*sgRNA |
| FT-BRI1R: | GCTATTTCTAGCTCTAAAACGAGATATCGTTATGACCCATTGGCCATAAGTAACCTTTAGAG |  |
| Atactin2F: | GCACCCTGTTCTTCTTACCG | PCR amplification of *Atactin2* |
| Atactin2R: | AACCCTCGTAGATTGGCACA |  |
| Cas9F: | AGCAGCCGACAAGAAGTACA | RT-PCR analysis of *Cas9* mRNA accumulation |
| Cas9R: | CGTCTGGTGTATCTTCTTCT |  |
| CLCrV-BF: | ATGTACAGTTTAAAGAGTAGACG | CLCrV-B virus detection |
| CLCrV-BR: | ATTATCCAATATAATCAAGGTCATAC |  |
| GUSF: | CGTGAAATCAAAAAACTCGACG | qRT-PCR analysis of *GUS* mRNA accumulation |
| GUSR: | GCCCGCATAATTACGAATATC |  |
