# Supplementary material for: Heritable gene editing using FT mobile guide RNAs and DNA viruses
Source: Plant Methods. 2021 Feb 17;17:20. doi: 10.1186/s13007-021-00719-4 (PMC7890912; doi:10.1186/s13007-021-00719-4)
Supplement: Supplementary file 2 — Additional file 2. The DNA sequence of targeted editing of AtGL2 by AtGL2-sgRNA. [file 13007_2021_719_MOESM2_ESM.docx]

**Additional Files**

**Heritable gene editing using *FT* mobile guide RNAs and DNA viruses**

Jianfeng Lei, Peihong Dai, Yue Li, Wanqi Zhang, Guantong Zhou, Chao Liu and Xiaodong Liu^*^

College of Agriculture, Xinjiang Agricultural University, Engineering Research Centre of Cotton, Ministry of Education, 311 Nongda East Road, Urumqi 830052, P.R. China

^*^Correspondence: Xiaodong Liu (xiaodongliu75@aliyun.com)

**Additional file 2.** The DNA sequence of targeted editing of *AtGL2* by *AtGL2*-sgRNA.

**
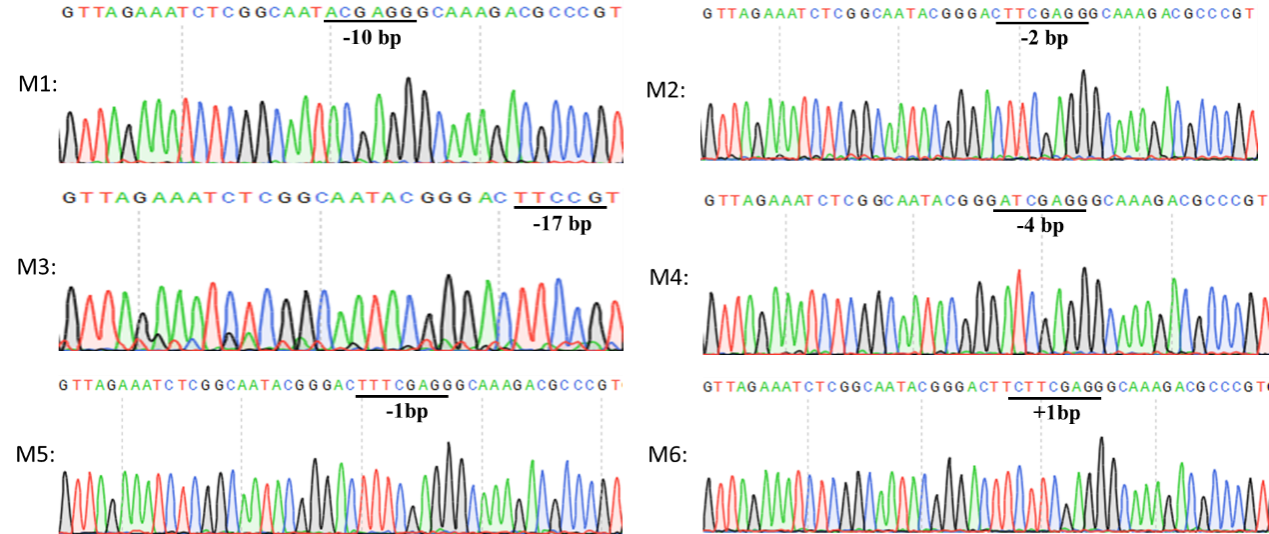
**
