# Supplementary material for: Heritable gene editing using FT mobile guide RNAs and DNA viruses
Source: Plant Methods. 2021 Feb 17;17:20. doi: 10.1186/s13007-021-00719-4 (PMC7890912; doi:10.1186/s13007-021-00719-4)
Supplement: Supplementary file 4 — Additional file 4. CLCrV-mediated targeted knockout of AtPDS. [file 13007_2021_719_MOESM4_ESM.docx]

**Additional Files**

**Heritable gene editing using *FT* mobile guide RNAs and DNA viruses**

Jianfeng Lei, Peihong Dai, Yue Li, Wanqi Zhang, Guantong Zhou, Chao Liu and Xiaodong Liu^*^

College of Agriculture, Xinjiang Agricultural University, Engineering Research Centre of Cotton, Ministry of Education, 311 Nongda East Road, Urumqi 830052, P.R. China

^*^Correspondence: Xiaodong Liu (xiaodongliu75@aliyun.com)

**Additional file 4.** CLCrV-mediated targeted knockout of *AtPDS*.

The *AtPDS* gene with obvious albino phenotype as the target [1], four AtU6-26::*AtPDS*-sgRNA vectors with different editing sites were constructed, and the four sgRNA vectors were recombined into CLCrV-A vector, co-transformed Cas9-OE *A. thaliana* leaves with CLCrV-B. 15-25 days after inoculation, *A. thaliana* leaves transformed with AtU6-26::*AtPDS*-sgRNA4 showed an incomplete albino phenotype (Fig. S4a), while the other three *AtPDS* editing sites did not have an albino phenotype. The *AtPDS* gene was further cloned and sequenced to verify whether mutations occurred. The *AtPDS* gene was amplified by PCR. After detection, the plants transformed with AtU6-26::*AtPDS*-sgRNA1, AtU6-26::*AtPDS*-sgRNA2 and AtU6-26::*AtPDS*-sgRNA3 did not have mutations. Since there was no suitable restriction site on the target site of AtU6-26::*AtPDS*-sgRNA4, we directly sequenced the PCR product and found that there was a multi-peak pattern in the target sequence region (Fig. S4b). It was the insertion or deletion of bases in the target region that causes base shifts. We cloned and sequenced the *AtPDS* gene (761 bp) of this plant, and the results showed that there were mutation types of deletion -1 bp and base substitutions (Fig. S4c).

**
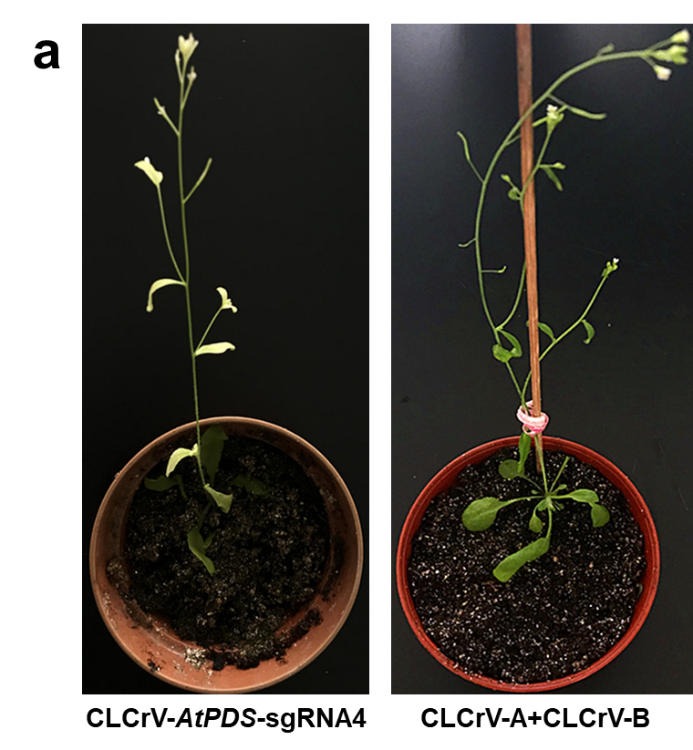

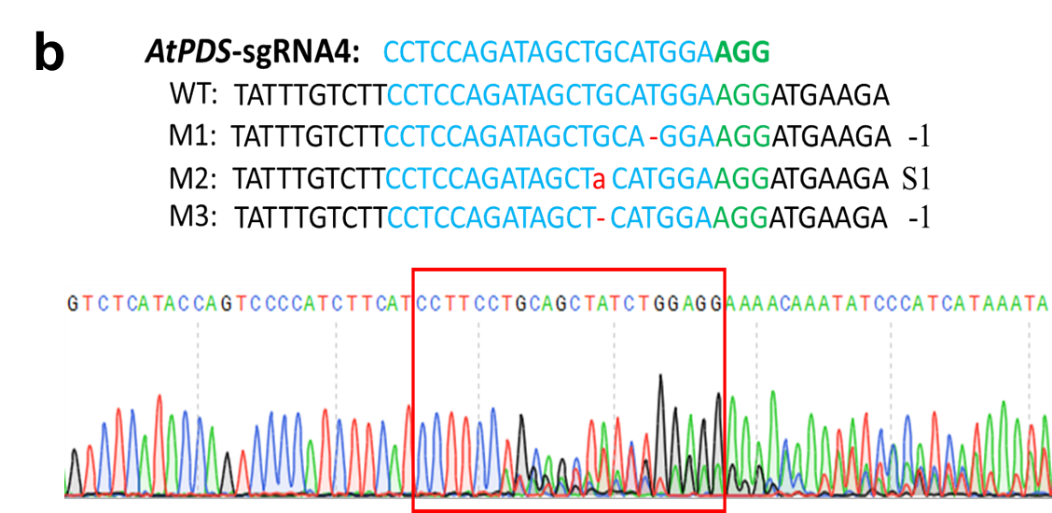
**


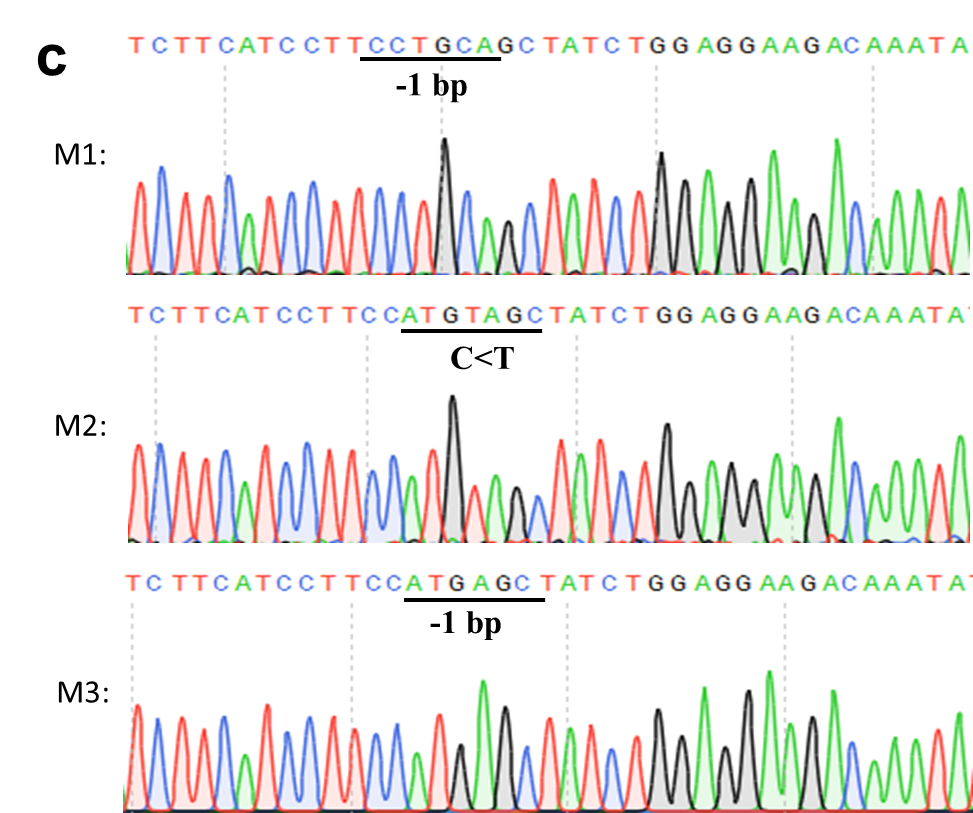


**Fig. S4** CLCrV-mediated targeted mutagenesis of *AtPDS* in *A. thaliana.* **a** Mutant phenotype of systemically infected Cas9-OE *A. thaliana* leaves at 15-25 days post-infiltration with CLCrV-*AtPDS*-sgRNA4, *A. thaliana* plants inoculated with CLCrV-A and CLCrV-B empty vectors served as a control. **b** Detection of *AtPDS*-sgRNA4 targeted mutations. Sequencing of the PCR product of *AtPDS* gene showed multiple peaks in the target sequence region (red box). **c** Cloning, sequencing and peak type analysis of *AtPDS* gene. Green color indicated the PAM sequence. Underline in blue indicated the target sequence. M indicated the mutation sequence. Deletions are shown as red dashes. Substitutions are denoted with red lowercase letters.
